# Combustion Synthesis and Photoluminescence Properties of Red-Emitting CaAlSiN_3_:Eu^2+^ Phosphor for White-LEDs

**DOI:** 10.3390/ma7127828

**Published:** 2014-12-05

**Authors:** Shyan-Lung Chung, Shu-Chi Huang

**Affiliations:** 1 Department of Chemical Engineering, National Cheng Kung University, Tainan 70101, Taiwan; E-Mail: n38981266@mail.ncku.edu.tw; 2 Advanced Optoelectronic Technology Center, National Cheng Kung University, Tainan 70101, Taiwan

**Keywords:** CaAlSiN_3_:Eu^2+^, nitridosilicate phosphor, LED lighting, combustion synthesis

## Abstract

A combustion synthesis method has been developed for synthesis of Eu^2+^ doped CaAlSiN_3_ phosphor and its photoluminescence properties were investigated. Ca, Al, Si, and Eu_2_O_3_ powders were used as the Ca, Al, Si and Eu sources. The addition of NaN_3_, NH_4_Cl and Si_3_N_4_ powders was found to increase significantly the product yield. These powders were mixed and pressed into a compact, which was then wrapped up with an igniting agent (*i.e.*, Mg+Fe_3_O_4_). The compact was ignited by electrical heating under a N_2_ pressure of ≤1.0 MPa. Effects of these experimental parameters on the product yield were investigated and a reaction mechanism was proposed. The synthesized CaAlSiN_3_:Eu^2+^ phosphor absorbs light in the region of 200–600 nm and shows a broad band emission in the region of 500–800 nm due to the 4f^6^5d^1^ → 4f^7^ transition of Eu^2+^. The sample doped with Eu^2+^ at the optimized molar ratio of 0.04 is efficiently excited by the blue light (460 nm) and generates emission peaking at ~650 nm with peak emission intensity ~106% of a commercially available phosphor, YAG:Ce^3+^(P46-Y3).The internal quantum efficiency of the synthesized phosphor was measured to be 71%, compared to 69% of the YAG:Ce^3+^ (P46-Y3).

## 1. Introduction

White light LED lighting has been replacing conventional lighting and becoming the next generation lighting device due to advantages such as energy efficiency, long lifetime, compactness, environmental friendliness and designable features [1,2,3,4]. Phosphors are essential materials for the fabrication of the LED lighting devices and their properties significantly affect the performance of the devices. Among various types of phosphor, the type with red emission has been considered to be the most urgent one to be developed for two main reasons: Its use can improve the color rendering of the currently commercialized LED lighting devices and conventional red phosphors (e.g., Sr_1−*x*_Ca_*x*_S:Eu^2+^ and Y_2_O_2_S:Eu^3+^) suffer from poor chemical stability and low quantum efficiency. In the past decade, a class of phosphors (*i.e.*, rare-earth doped nitridosilicates) were discovered and shown to be ideal for application in LED lighting due to their superior properties such as high quantum efficiency, long wavelength (red) emission and high thermal and chemical stability [5]. Two major types of nitridosilicate have been developed as the host lattices for the nitridosilicate phosphors, namely alkaline-earth silicon nitrides (M_2_Si_5_N_8_, M = Ca, Sr and Ba, often referred to as 2-5-8 phosphors) and alkaline-earth aluminum silicon nitrides (MAlSiN_3_, M = Mg, Ca and Sr, often referred to as 1-1-1-3 phosphors). Many methods have been developed for the synthesis of nitridosilicate phosphors including [5] solid state reaction (SSR) [6], carbothermal reduction and nitridation (CRN) [7], gas pressure sintering [8], reaction between metals and silicon diimide [9], gas reduction and nitridation [10], high pressure ammonothermal method [11], spark plasma sintering [12], alloy-nitridation [13], nitrate reduction [14] and combustion synthesis (SHS) [15]. However, many of these methods utilize costly and oxygen- or moisture-sensitive chemicals as the starting materials, and most of the methods are carried out under severe synthesis conditions (e.g., high temperatures, high pressures and long reaction time). These problems may hinder the practical application of the methods. Further studies in the development of the synthesis method are thus needed so that the nitridosilicate phosphors can be produced under easier synthesis conditions with low production costs, thus boosting the practical applications of LED lighting.

In our previous study [16], a combustion synthesis method was developed for the synthesis of Eu^2+^-doped Ca_2_Si_5_N_8_ phosphor: Ca, Si and Eu_2_O_3_ powders were used as the Ca, Si and Eu sources and NaN_3_, NH_4_Cl and Si_3_N_4_ were added to enhance the product yield. The synthesis reaction was triggered by the combustion of an igniting agent, which wrapped up the reactant compact. A product yield of ~71% was obtained under a N_2_ pressure of 0.7 MPa. In addition to easy handling of the reactants and a low N_2_ pressure required (~0.7 MPa), the method developed possesses many other advantages including simple and inexpensive equipment required, relatively low cost of the reactants, a fast reaction and short processing time, potential capability for mass production and possibly low production costs.

When comparing with 2-5-8 phosphors, 1-1-1-3 phosphors were found to have many superior properties [17,18,19] such as an even higher thermal stability, a longer emission wavelength and a higher quantum efficiency. However, the 1-1-1-3 phosphors were also found to require even more severe conditions for their synthesis (as compared to that of the 2-5-8 phosphors) [20]. In the present study, the combustion synthesis method developed in our previous study [16] was tested and modified for the synthesis of CaAlSiN_3_:Eu^2+^ phosphor. Reactants were chosen to be those which could be handled in ambient air and the process was designed to be carried out under a low N_2_ pressure limited to our SHS reactor (≤1.0 MPa). The development of the process and the effects of process parameters on the product yield will be described and a reaction mechanism will be proposed. The luminescent properties of the synthesized phosphor will also be reported and discussed.

## 2. Results and Discussion

### 2.1. Optimum Synthesis Condition and Combustion Phenomena

**Figure 1:**
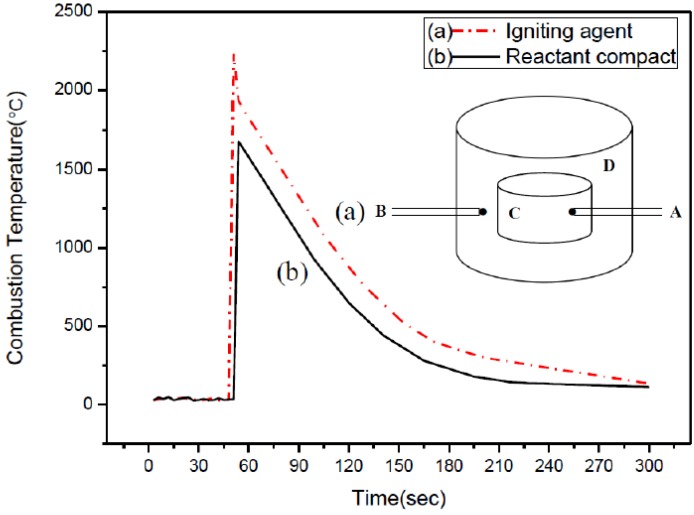
Typical temperature-time histories during combustion reaction.

After a series of experiments (some of which will be described later), an optimum synthesis condition (achieving the highest product yield) was found to be that the reactant compact had a composition of Ca:Al:Si:NaN_3_:NH_4_Cl:Si_3_N_4_:Eu_2_O_3_ = 1−2*x*:1:0.25:3.5:0.6:0.25:*x* and that the combustion synthesis reaction was carried out under a N_2_ pressure of 0.9 MPa (all the reactant compositions were expressed as molar ratios in this work). Under such an optimum synthesis condition, it was observed that the igniting agent was ignited when the heating power had been turned on for ~10 s and subsequently, the combustion wave propagated down the compact. During propagation of the combustion wave, visible radiation was observed to be emitted from the interior indicating the occurrence of the synthesis reaction of the reactant compact (this visible radiation was observed to last for ~5 s). Figure 1 shows typical temperature-time histories measured by thermocouples A and B. Both profiles show abrupt increases in temperature, which were caused by heat transfer from the combustion waves (when approaching the points of measurement) and also by the onset of their own combustion. The abrupt increase in profile (b) occurred several seconds after that of profile (a), indicating that the combustion of the reactant compact was caused by heating by the combustion of the igniting agent. The maximum combustion temperatures of the igniting agent and the reactant compact were measured to be ~2230 °C and ~1730 °C, respectively.

**Figure 2:**
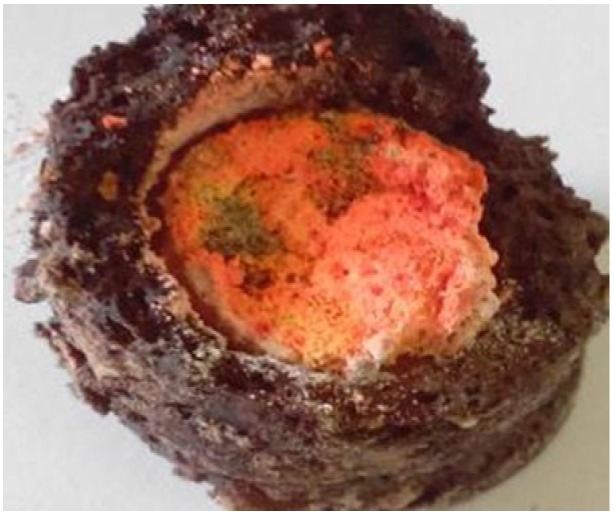
A photograph of the as-synthesized product obtained under the optimum synthesis condition. The outer layer (black in color) is the combustion product of the igniting agent and the inside portion (red in color) is the synthesized phosphor.

Figure 2 is a photograph of the as-synthesized product obtained under the optimum synthesis condition (*i.e.*, Ca:Al:Si:NaN_3_:NH_4_Cl:Si_3_N_4_:Eu_2_O_3_ = 0.92:1:0.25:3.5:0.6:0.25:0.04 under a N_2_ pressure of 0.9 MPa). As can be seen, the as-synthesized product was highly porous. The outer layer (black in color) is the combustion product of the igniting agent and the inside portion (red in color) is the synthesized phosphor. After removing the outer layer, the product was ground by using a mortar and pestle for ~3 min to powder for characterization.

**Figure 3:**
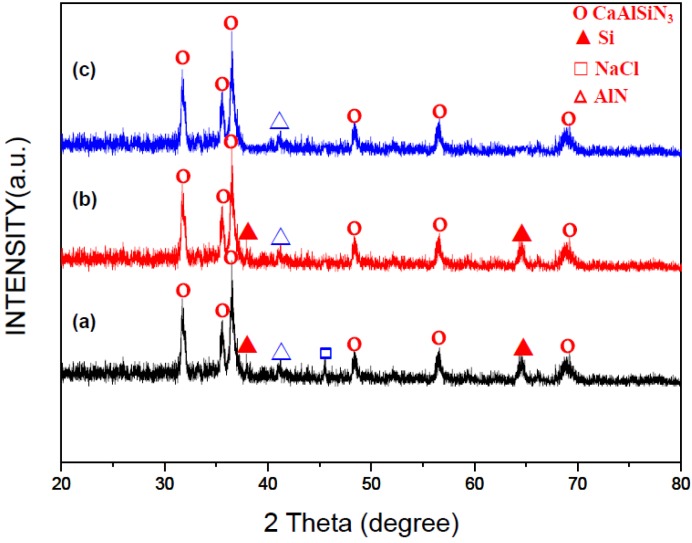
XRD patterns of the products: (**a**) as-synthesized; (**b**) after washing with water; and (**c**) after washing with water and an acid.

Figure 3a is an XRD pattern of the as-synthesized product obtained under the optimum synthesis condition. As can be seen, in addition to the phase of CaAlSiN_3_:Eu^2+^ (JCPDS No.39-0747), AlN (JCPDS No. 88-2363), NaCl (JCPDS No. 88-2300) and residual Si (JCPDS No. 79-0613) were also detected. As mentioned previously [16], NaCl could be removed by washing the product with water and residual Si could be removed by washing the product with an acid. Figure 3b,c are the XRD patterns of the products after these washings.

**Figure 4:**
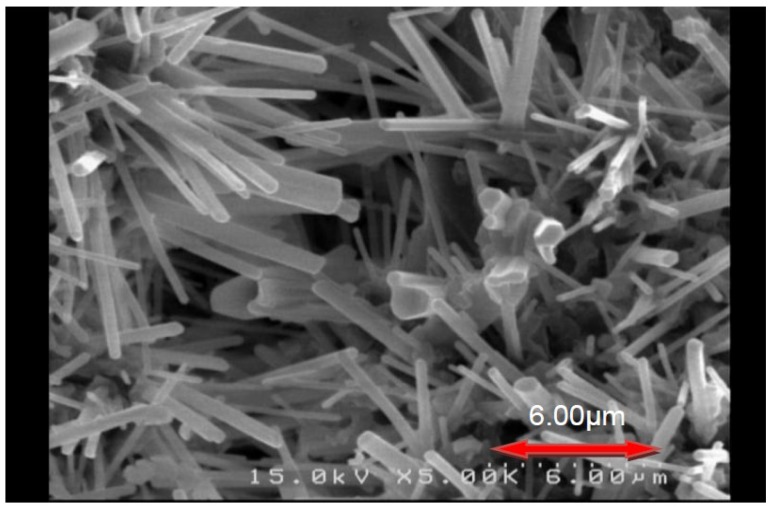
A typical SEM photograph of the product (Ca:Al:Si:NaN_3_:NH_4_Cl:Si_3_N_4_:Eu_2_O_3_ = 0.92:1:0.25:3.5:0.6:0.25:0.04) after grinding and removing NaCl and the residual Si.

The powder thus obtained (after removing NaCl and the residual Si) appeared red in color and most of the particles were observed (see Figure 4) to be columnar in shape (with a diameter ranging from 0.1 to 0.8 μm and a length ranging from 5 to 10 μm). The average particle size (*d*_50_) was measured by the particle size analyzer to be 8.8μm

### 2.2. Effects of Process Parameters on Product Yield

#### 2.2.1. Effects of NH_4_Cl and NaN_3_

**Figure 5:**
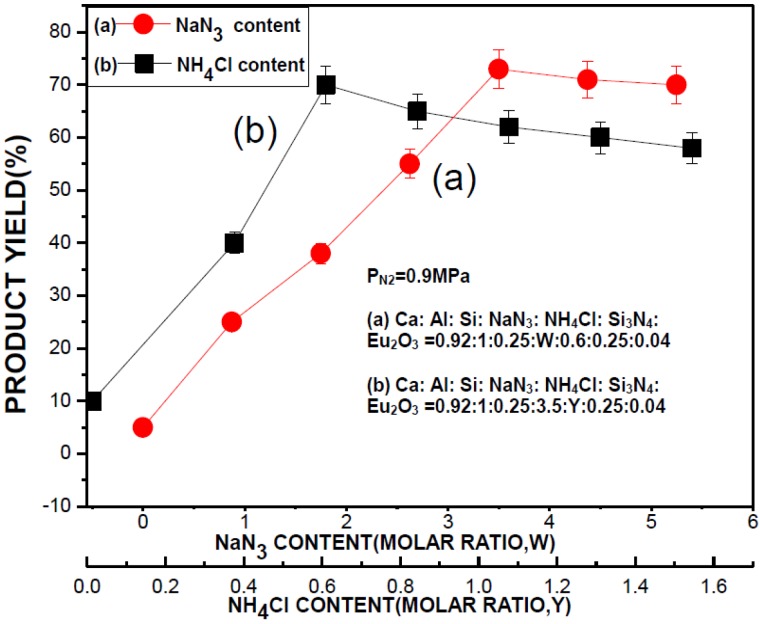
Effects of (**a**) NaN_3_ and (**b**) NH_4_Cl contents on the product yield.

Figure 5 shows the effects of NaN_3_ and NH_4_Cl contents on the product yield. The contents of other reactants and the N_2_ pressure were kept the same as the optimum synthesis condition. As can be seen, the product yield was very low (5% or 10%, respectively) when no NaN_3_ or NH_4_Cl no was added. The product yield increases with increasing NH_4_Cl content to a maximum of ~71% (at a molar ratio of 0.6) and begins to decrease with further increase in NH_4_Cl content. In the case of adding NaN_3_, the product yield is seen in Figure 5 (curve (a)) to increase with increasing NaN_3_ content to ~74% (at a molar ratio of 3.5) and becomes relatively unaffected with further increase in the NaN_3_ content. In our previous studies on the combustion synthesis of Si_3_N_4_ [21]. NH_4_X (X = F, Cl, Br or I with NH_4_Cl being the most effective) and NaN_3_ were found necessary for the formation of Si_3_N_4_ and a reaction mechanism was proposed: Si was considered to react with HX (produced by thermal decomposition of NH_4_X), converting itself to more reactive species, SiX*_x_*. SiX*_x_* was then reduced by Na vapor (produced by thermal decomposition of NaN_3_) and subsequently, nitridation of Si takes place, resulting in the formation of Si_3_N_4_. NH_4_X and NaN_3_ were thus referred to as catalytic and reducing agents, respectively. In the present study, the formation of CaAlSiN_3_:Eu^2+^ is believed to follow a similar mechanism. Both NH_4_Cl and NaN_3_ are thus required to achieve high products.

**Figure 6:**
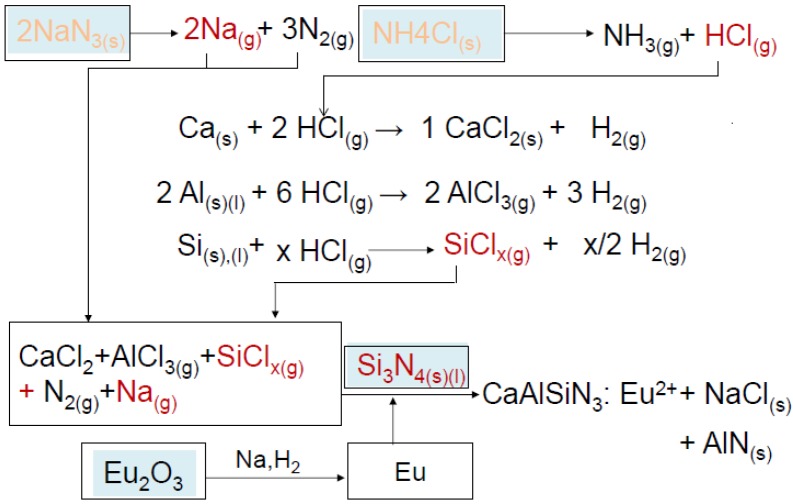
The proposed reaction mechanism.

In the present study, Ca, Al were also considered to react with HCl forming CaCl_2_ and AlCl_3_. As illustrated in Figure 6, SiCl*_x_*, CaCl_2_, and AlCl_3_ were then reduced by Na vapor and subsequently, nitridation of the reduced species takes place, forming the host lattice. In the meantime, Eu_2_O_3_ may be reduced by Na vapor or H_2_ (generated by reactions of Si and HCl, see Figure 6) and incorporates into the host lattice forming the product phase, CaAlSiN_3_:Eu^2+^.

When the NH_4_Cl content is low (at a molar ratio of <0.6), increasing the NH_4_Cl content (while keeping the contents of other reactants constant) increases the concentration of HCl, promoting the formation of the chlorides of Si, Ca, and Al. Formation of CaAlSiN_3_:Eu^2+^ is therefore enhanced, resulting in a higher product yield. When the NH_4_Cl content was higher than a molar ratio of 0.6, a violent gas evolution occurred during the combustion. Much powder was thus thrown out of the reactant compact and deposited on the reactor walls. (This powder was collected and identified by XRD analysis to be mostly CaAlSiN_3_:Eu^2+^). The product yield thus decreases with increasing NH_4_Cl content. When increasing the NaN_3_ content, the concentration of Na vapor is increased, promoting the reduction and thus the nitridation reactions. The product yield thus increases with increasing NaN_3_ content. As the NaN_3_ content is further increased (with a molar ratio >3.5), the product yield is relatively unaffected because a sufficient concentration of Na has been generated.

#### 2.2.2. Effects of Si_3_N_4_

**Figure 7:**
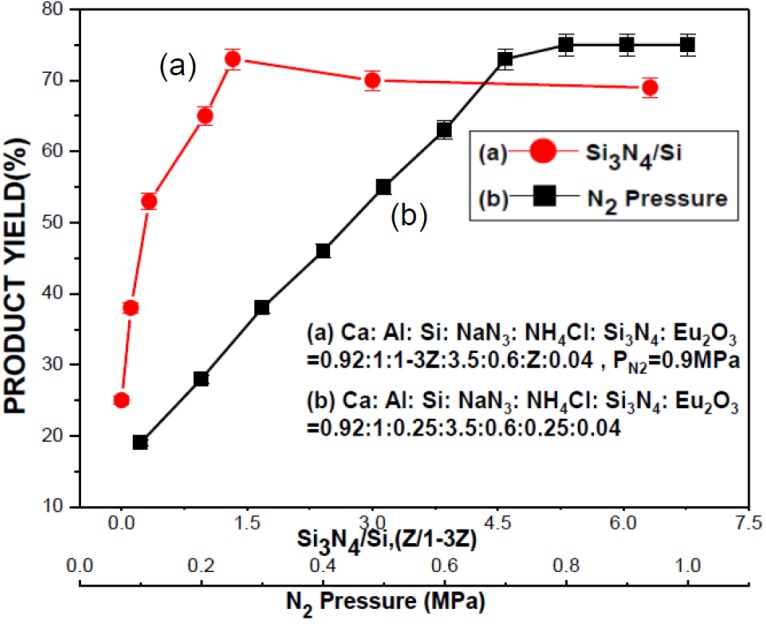
Effects of (**a**) Si_3_N_4_ Content (expressed as the molar ratio of Si_3_N_4_/Si) and (**b**) N_2_ pressure on the product yield.

As mentioned previously, the product yield could be significantly increased by adding a proper amount of Si_3_N_4_ to the reactant compact. In studying the effect of Si_3_N_4_, the total amount of Si (*i.e.*, the Si powder and the Si contained in the Si_3_N_4_ powder) was kept constant at a molar ratio of 1 (being equal to that in the optimum synthesis condition) and the amount of Si_3_N_4_ was expressed as the molar ratio of Si_3_N_4_ to Si (*i.e.*, Si_3_N_4_/Si). Curve (a) in Figure 7 shows the effects of Si_3_N_4_/Si on the product yield. As can be seen, the product yield increases with increasing Si_3_N_4_/Si to a maximum of ~74% at Si_3_N_4_/Si = 1.33 and begins to decrease with further increase in Si_3_N_4_/Si.

When the reactant compact was prepared without addition of Si_3_N_4_ (*i.e.*, Si_3_N_4_/Si = 0), many large and spherical particles (with diameters up to 20 μm) were observed (with an SEM) on fractured surfaces of the as-synthesized products. These particles were identified by elementary analysis to be Si particles and they became smaller and were reduced in number when increasing Si_3_N_4_/Si. These spherical Si particles were believed to be formed by melting and coalescence of the Si particles added when preparing the reactant compact. Because formation of large and spherical particles decreases the surface area for solid (or liquid)-gas reactions, coalescence hinders the nitridation reaction, thus lowering the product yield. Coalescence of Si is believed to be reduced at the presence of Si_3_N_4_ particles because the molten Si can spread over the Si_3_N_4_ particles due to capillary force. This capillary spreading increases the surface area for solid (or liquid)-gas reaction. The product yield thus increases with increasing Si_3_N_4_/Si (in the range of Si_3_N_4_/Si < 1.33, see Figure 7). As the Si_3_N_4_/Si is further increased, the combustion temperature begins to decrease because of the cooling effect of a high content of Si_3_N_4_ and also a less amount of Si available for combustion, thus resulting in a decrease in the product yield. As can be seen in Figure 3, Si_3_N_4_ was not detected in the product by XRD. The Si_3_N_4_ added to the reactant compact was thus believed to be converted completely to the product phase (CaAlSiN_3_:Eu^2+^).

#### 2.2.3. Effect of N_2_ Pressure

The effect of nitrogen pressure on the product yield is shown as curve (b) in Figure 7. (The reactant composition was kept the same as the optimum synthesis condition, *i.e.*, Ca:Al:Si:NaN_3_:NH_4_Cl:Si_3_N_4_:Eu_2_O_3_ = 0.92:1:0.25:3.5:0.6:0.25:0.04). As can be seen, the product yield increases with increasing nitrogen pressure to ~75% at nitrogen pressure of 0.9 MPa and becomes relatively unaffected at higher N_2_ pressures. The amount of nitrogen available to the combustion synthesis reaction increases with increasing nitrogen pressure. A higher N_2_ pressure thus results in a higher reaction rate, and thus a higher product yield (in the range of ≤0.9 MPa, see Figure 7). As a sufficient amount of N_2_ is supplied, the reaction rate and thus the product yield become relatively unaffected with further increase in the N_2_ pressure (>0.9 MPa, see Figure 7).

### 2.3. Photoluminescence Properties of CaAlSiN_3_:Eu^2+^ Phosphor

**Figure 8:**
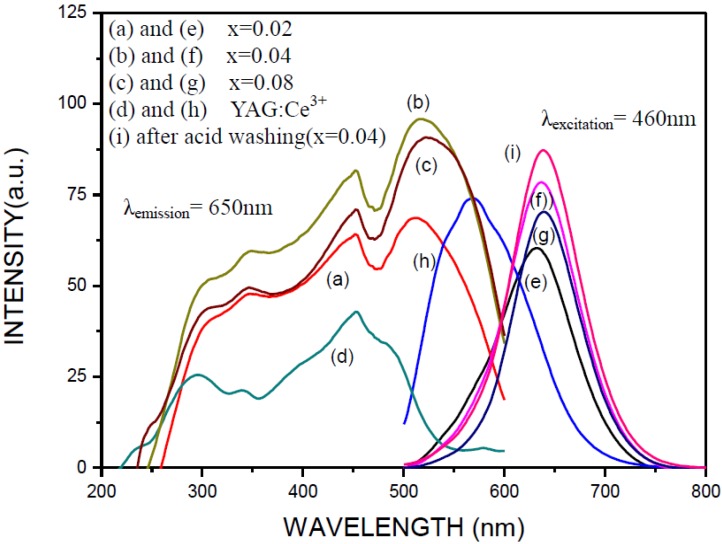
The excitation ((**a**), (**b**) and (**c**)) and emission ((**e**), (**f**), (**g**) and (**i**)) spectra of the phosphor synthesized in this study and similar spectra of YAG: Ce^3+^ phosphor (P46-Y3) are also shown ((**d**) and (**h**)) for comparison.

Curves (a), (b), and (c) and (e), (f), and (g) in Figure 8 are the excitation and emission spectra (λ_excitation_ = 460 nm), respectively, of the phosphor powders (after removing NaCl and the residual Si) synthesized under the optimum synthesis condition, that is Ca:Al:Si:NaN_3_:NH_4_Cl:Si_3_N_4_:Eu_2_O_3_ = 0.92:1:0.25:3.5:0.6:0.25:*x* (*x* = 0.02, 0.04, and 0.08) and N_2_ pressure =0.9 MPa. As can be seen, the intensities of the excitation and emission both increase when the Eu_2_O_3_ molar ratio (*x*) is increased from 0.02 to 0.04, but both decrease when the Eu_2_O_3_ molar ratio is further increased to 0.08. The excitation spectra all consist of two main absorption bands: The one in the range of 200–350 nm is ascribed to the host lattice excitation due to transition from the valence to the conduction band. The other excitation band being in the range of 350–600 nm is assigned to the 4f^7^ → 4f^6^5d^1^ transition of the Eu^2+^ ion. All the emission spectra show a single broad band emission in the range of 500–800 nm (which are similar to the literature) and are attributed to the allowed 4f^6^5d^1^ → 4f^7^ transition of the Eu^2+^ ion. For comparison, similar spectra of a commercially available YAG:Ce^3+^ phosphor (P46-Y3) were also measured (under the same measurement conditions as those for the synthesized phosphors) and are shown as curves (d) and (h) in Figure 8. As can be seen, the peak emission intensity of the synthesized phosphor is ~106% of that of YAG:Ce^3+^ at 0.04 of the Eu_2_O_3_ molar ratio. Internal quantum efficiencies (IQE) of the synthesized phosphor and the commercial YAG:Ce^3+^ (P46-Y3) phosphor were also measured (with λexcitation = 460 nm) to be 71% and 69%, respectively. By comparison, the emission wavelength range measured in the present study is similar to those reported in many other studies [22,23,24,25] and the emission intensity is comparable to that reported by Piao *et al.* [26] for a similar phosphor.

**Figure 9:**
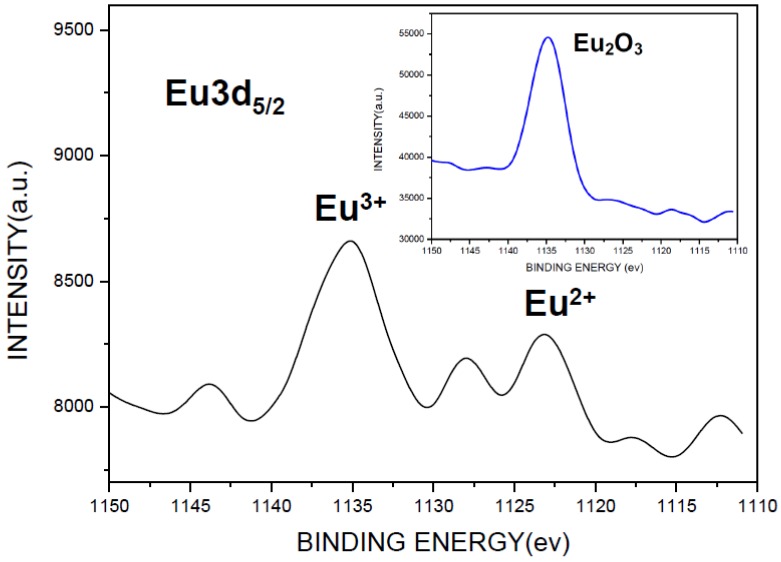
XPS spectra in the Eu 3d_5/2_ region of reactant Eu_2_O_3_ (inset) and the synthesized product.

As can be seen in Figure 8, the emission intensity depends strongly on the Eu_2_O_3_ content in the reactant compact. A question thus arises that whether or not the added Eu_2_O_3_ is completely incorporated into the host lattice. To clarify this problem, XPS was employed to identify the chemical state of the Eu. As shown in Figure 9, in addition to the characteristic peak of Eu^2+^ (at 1123 eV [27,28,29,30]), the characteristic peak of Eu^3+^ (at 1135 eV [27,28,29,30]) was also detected for the synthesized product, which is located at the same position as that of the Eu_2_O_3_ added to the reactant (the inset in Figure 9). Since sharp line emission peaks, characteristic of ^5^D_0_ → ^7^F_J_ transition of Eu^3+^ [31], were not observed (see Figure 8), Eu^3+^ is not considered to be incorporated into the host lattice. These results thus indicate that a certain fraction of Eu_2_O_3_ was unreacted and contained in the product. Therefore, the actual concentration of Eu^2+^ in the host lattice is believed to be lower than that calculated based on the reactant composition. The unreacted Eu_2_O_3_ contained in the product can be removed by washing the product with hydrochloric acid. As shown in Figure 8 (curve (i)), the emission intensity of the synthesized phosphor increases slightly after this acid washing.

**Figure 10:**
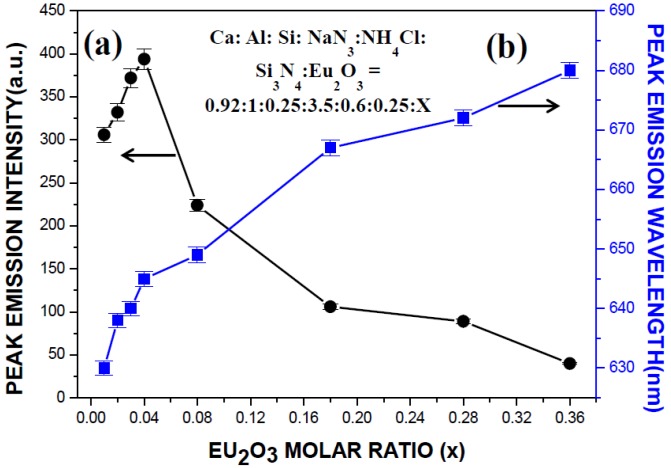
Dependence of (**a**) peak emission intensity and (**b**) peak emission wavelength on the Eu_2_O_3_ molar ratio, *x*.

The dependence of the peak emission intensity and wavelength on the Eu_2_O_3_ molar ratio (*i.e.*, the *x* in Ca:Al:Si:NaN_3_:NH_4_Cl:Si_3_N_4_:Eu_2_O_3_ = 0.92:1:0.25:3.5:0.6:0.25:*x*) is shown in Figure 10. The peak emission intensity increases with increasing Eu_2_O_3_ molar ratio to a maximum at the molar ratio of 0.04 and begins to decrease with further increase in the molar ratio. At the same time, the emission band shows a redshift: The peak emission wavelength increases from 630 to 680 nm as the Eu_2_O_3_ molar ratio is increased from 0.01 to 0.18. Although a certain fraction of Eu_2_O_3_ was found unreacted, the Eu^2+^ concentration in the lattice of the product phase increases with increasing Eu_2_O_3_ molar ratio in the range of this study. This is seen in Figure 10 where the peak emission wavelength increases continuously with increasing Eu_2_O_3_ molar ratio. The decrease in peak emission intensity beyond a critical Eu^2+^ concentration (where the Eu_2_O_3_ molar ratio is 0.04, see Figure 10) is considered to be due to concentration quenching, which occurred when the interatomic distance among the Eu^2+^ ions is shortened, causing frequent energy transfer among them [32]. The redshift can be due to an increase in probability of the energy transfer of Eu^2+^ from higher to lower levels of 5d, thus increasing the emission intensity in the long wavelength region [33]. As pointed out in many other studies [34,35,36], the redshift may also be explained by reabsorption due to overlapping of the excitation and the emission spectra. Note that the overlapping of the excitation and the emission spectra is seen in Figure 8 to increase with increasing Eu_2_O_3_ molar ratio. In addition, the incorporation of Eu^2+^ on Ca^2+^ site results in distortion of the lattice, which may also contribute to the redshift of the emission [37].

## 3. Experimental Section

### 3.1. Development of the Process

As mentioned previously, this study is aimed at developing a combustion synthesis process for synthesis of CaAlSiN_3_:Eu^2+^ phosphor at low N_2_ pressures by using reactants which can be handled in ambient air. Based on this thought, Ca, Al, Si and Eu_2_O_3_ powders were chosen as the reactants serving as the sources of Ca, Al, Si, and Eu in CaAlSiN_3_:Eu^2+^ phosphor. However, it was found that the compact of the mixture of these powders (referred to as reactant compact hereafter) could not be ignited under a N_2_ pressure limited to the reactor used in the present study (*i.e.*, ≤1.0 MPa). This was considered to be due to the low reactivity of Ca, Si with respect to N_2_ at pressures of ≤1.0 MPa. The reactant compact was thus wrapped up with an igniting agent (*i.e.*, the mixture of Mg and Fe_3_O_4_ powders), which can undergo a high exothermic combustion reaction, thus heating up quickly the reactant compact and causing its combustion reaction to occur. Besides, it was found that the product yield could be significantly enhanced by adding proper amounts of NaN_3_, NH_4_Cl and Si_3_N_4_ simultaneously. In the combustion synthesis process developed in the present study, the reactant compact is thus composed of Ca, Al, Si, Eu_2_O_3_, NaN_3_, NH_4_Cl and Si_3_N_4_ and the reactant compact is wrapped up with an igniting agent.

### 3.2. Description of Experiment

Listed in Table 1 are the characteristics of the reagents used in this study. Silicon, aluminum, calcium, europium oxide, sodium azide , ammonium chloride and silicon nitride powders were thoroughly mixed in the desired proportions and then pressed into cylindrical compacts (*i.e.*, the reactant compacts) with 17 mm in diameter and ~16 mm in length. A stainless steel die with two plungers was used and a pressure of 300 MPa was uniaxially applied in forming the compacts. The reactant compact thus obtained was placed in a larger die and the space between the compact and the die was filled with an igniting agent (*i.e.*, a mixed powder of Mg and Fe_3_O_4_ at 4:1 molar ratio). By uniaxially applying a pressure of 300 MPa, the reactant compact was wrapped up with the igniting agent, which was a larger cylindrical compact (referred to as a wrapped compact, see the insert in Figure 1) with 30 mm in diameter and ~30 mm in length.

**Table 1:** Characteristics of the reagents used in this study.

Reagent	Particle Size (um)	Purity (%)	Source
Si	1–5	99	Alfa Aesar
Ca	<963	99.5	Alfa Aesar
Eu_2_O_3_	2.5–6	99.99	Seedchem Company
NaN_3_	*d*_50_ ≅ 150	99	Johnson Matthey
NH_4_Cl	–	99	Panreac
Al	*d*_50_ ≅ 3	99	First Chemical Work
α-Si_3_N_4_	*d*_50_ ≅ 10	95	Alfa Aesar
Mg	*d*_50_ ≅ 20	99	Nihon Shiyaku
Fe_3_O_4_	*d*_50_ ≅ 102	99	Nihon Shiyaku
N_2_	Gas	99	Yun Shan

The combustion synthesis reactor used in this study has been described and shown schematically in our previous studies [38,39,40,41,42] and thus is not repeated here. The wrapped compact was placed on a height adjustable stage which was adapted so that the top surface of the compact was about 5 mm below the tungsten heating coil. The reactor was evacuated to 65 Pa by flushing with nitrogen between the evacuations. After the evacuation, the reactor was backfilled with nitrogen to the desired pressures. The combustion reaction was ignited by heating the top surface of the compact for ~10 s by applying an electrical power of ~1 KW to the heating coil.

Variation of temperature during combustion reaction was measured by using 0.13 mm diameter W-5%Re-W-26%Re thermocouples. The thermocouples, insulated with 1.2 mm diameter alumina tubes, were inserted into the compact at appropriate depths by first drilling holes. As shown by the insert in Figure 1, the temperature variation of the reactant compact was measured by thermocouple A while that of the igniting agent was measured by thermocouple B. After combustion, the igniting agent was converted to MgO+Fe, which was loosely attached to the interior product. The interior product could thus be easily separated from the combustion product of the igniting agent. The as-synthesized products contained byproducts, AlN and NaCl, and small amounts of unreacted Si. NaCl could be removed by washing the products with water and unreacted Si could be removed by washing the products with an acid (e.g., a mixed solution of 48 wt% HF_(aq)_ and 68 wt% HNO_3(aq)_ at a volume ratio of 5:1 as used in this study). The product yield (defined as the percentage of the Si converted to the product) was obtained from the weight ratio of the Si contained in the product (being assumed to be all CaAlSiN_3_:Eu^2+^ after removal of NaCl and unreacted Si) to that in the reactant compact (including the Si powder and the Si in Si_3_N_4_). The crystalline phase of the product was identified by X-ray diffraction (XRD, DMAX-200/PC, Rigaku, Tokyo, Japan) using Cu Kα radiation operating at 40kV and 30 mA. The data was collected at a scanning speed of 10°/min between 20° and 80° with a scanning step of 0.01° in 2θ. The morphology of the product was analyzed with a scanning electron microscope (S-4100, Hitachi, Tokyo, Japan). The particle size distribution was measured by a particle size analyzer (LA-950, Horiba, Tokyo, Japan). The excitation and emission spectra were measured at room temperature using a fluorescent spectrophotometer (FP-6600, Jasco, Tokyo, Japan) with a 150 W xenon lamp at a scanning speed of 125 nm/min. The internal quantum efficiency [43] was measured using a fluorescent spectrometer (F-7000; Hitachi, Tokyo, Japan) with an integrating sphere system (5JO-0148; Hitachi, Tokyo, Japan). The light from a 200 W xenon lamp was used as the excitation source at a scanning speed of 240 nm/min. The chemical states of elements were measured by X-ray photoelectron spectroscopy with an Al Kα X-ray source (ESCA-LAB 250, VG Scientific, London, UK).

## 4. Conclusions

A combustion synthesis method has been developed for synthesis of CaAlSiN_3_:Eu^2+^ phosphor at low N_2_ pressures by using reactants which can be handled in ambient air. The synthesis reaction is triggered by the combustion of an igniting agent, which is ignited by heating for ~10 s with an electrical power of ~1 KW and it takes ~10 s for the synthesis reaction to complete. In addition to easy handling of the reactants and a low N_2_ pressure required (~0.9 MPa), the method developed in the present study possesses many other advantages including simple and inexpensive equipment required, relatively low cost of the reactants, a fast reaction and short processing time, potential capability for mass production and possibly low production costs. The synthesized CaAlSiN_3_:Eu^2+^ phosphor absorbs light in the region of 200–600 nm and shows a broad band emission in the region of 500–800 nm. The sample doped with Eu^2+^ at the optimized molar ratio of 0.04 is efficiently excited by the blue light (460 nm) and generates emission peaking at ~650 nm with peak emission intensity exceeding that of the YAG:Ce^3+^ (P46-Y3) phosphor by ~6%. The internal quantum efficiency of the synthesized phosphor was measured to be 71%, compared to 69% of the YAG:Ce^3+^ (P46-Y3).With further improvement, the combustion synthesis method developed in the present study may be potentially applied for industrial production of phosphors for application in white LED lighting.
